# Supplementary material for: Health care seeking patterns of rifampicin-resistant tuberculosis patients in Harare, Zimbabwe: A prospective cohort study
Source: PLoS One. 2021 Jul 16;16(7):e0254204. doi: 10.1371/journal.pone.0254204 (PMC8284678; doi:10.1371/journal.pone.0254204)
Supplement: Attachment — Submitted filename: Responses to reviewers_02March2021.docx [file pone.0254204.s002.docx]

Below are our responses to the Editor and reviewers’ comments. Our comments and responses are in **bold font.**

1. Please ensure that your manuscript meets PLOS ONE's style requirements, including those for file naming. The PLOS ONE style templates can be found at:

https://journals.plos.org/plosone/s/file?id=wjVg/PLOSOne_formatting_sample_main_body.pdf https://journals.plos.org/plosone/s/file?id=ba62/PLOSOne_formatting_sample_title_authors_affiliations.pdf

**Thank you, we have taken these guidelines into consideration in our revised manuscript.**

2. Please address the following:
- Please include additional information regarding the survey or questionnaire used in the study and ensure that you have provided sufficient details that others could replicate the analyses. For instance, if you developed a questionnaire as part of this study and it is not under a copyright more restrictive than CC-BY, please include a copy, in both the original language and English, as Supporting Information. In addition, please include further details of the development and validation of this tool.
- Please refer to any sample size calculations performed prior to participant recruitment. If these were not performed please justify the reasons. Please refer to our statistical reporting guidelines for assistance (<https://journals.plos.org/plosone/s/submission-guidelines.#loc-statistical-reporting>).

**The study questionnaire was not a validated tool, and is attached as Supporting Information. We undertook convenience sampling, hence no power or sample size calculations were done prior to the study.**

3.We note that you have indicated that data from this study are available upon request. PLOS only allows data to be available upon request if there are legal or ethical restrictions on sharing data publicly. For information on unacceptable data access restrictions, please see <http://journals.plos.org/plosone/s/data-availability#loc-unacceptable-data-access-restrictions>.

**We have provided a copy of our data and study questionnaires as requested.**

In your revised cover letter, please address the following prompts:

a) If there are ethical or legal restrictions on sharing a de-identified data set, please explain them in detail (e.g., data contain potentially identifying or sensitive patient information) and who has imposed them (e.g., an ethics committee). Please also provide contact information for a data access committee, ethics committee, or other institutional body to which data requests may be sent.

b) If there are no restrictions, please upload the minimal anonymized data set necessary to replicate your study findings as either Supporting Information files or to a stable, public repository and provide us with the relevant URLs, DOIs, or accession numbers. Please see http://www.bmj.com/content/340/bmj.c181.long for guidelines on how to de-identify and prepare clinical data for publication. For a list of acceptable repositories, please see <http://journals.plos.org/plosone/s/data-availability#loc-recommended-repositories>.

**We have uploaded a copy of our anonymised dataset as per the PLOS ONE guidelines.**

We will update your Data Availability statement on your behalf to reflect the information you provide.

Review Comments to the Author

Reviewer 1

Reviewer #1: Abstract

Page. 2, L25: are major impediments

**Corrected**

Page. 2, L28: using s prospective cohort study design

**corrected**

More information on data collection instruments, analysis etc. are required in the methods section.

**Thank you for this comment. We have added the following statement to the methods section under “Data collection”, Page 5:**

**“Data were collected using a mixed methods approach”.**

Page. 2, L33-34 should come first in the presentation of results…..

**corrected**

Page. 2, L29: The socio-demographic characteristics of the participants is missing. It is important for the subject under study

**Thank you for this comment. We only reported the key findings of the study in the abstract due to the word limit. However, socio-demographic data have been included in the main results section of the paper (Page 7) as suggested by the reviewer as follows:**

**“More than 70% (48/68) of study participants were female, mainly from the economically active age group (median 34 years, IQR 29-42 years); with more than 73%, (n=50/68) educated up to secondary level.”**

Introduction
Page. 3, L50-53: Consider making 2 sentences for better coherence**:**

**corrected**

Page. 3, L53: Remove ‘due to’:

**corrected**

Page. 3, L54: add ‘also’ after may:

**corrected**

Page. 3, L56: Change from…. in the community, which may TO …. in the community and this could….. MAY has been used severally in that paragraph-

**corrected**

Page. 3, L59: add the before availability-

**corrected**

Page. 3, L71: Study area/setting missing-

**corrected**

Page. 4, L87-92: Consider breaking long sentences-

**corrected**

Page. 4, L87, 96: Source of the questionnaire and definitions lacking. More information needed

**Thank you for this comment. We have added the following detail (pages 4-5) to the methods section with regards to the questionnaire used.**

**“Data were collected using a mixed methods approach via an open-ended healthcare-seeking behaviour questionnaire designed to capture both quantitative and qualitative responses through patient interviews. The open-ended questionnaire allowed respondents to provide detailed responses on their health seeking pathways and reasons for delay in seeking care.”**

Results
Page. 5, L122: More information on this main study is needed in the preceding sections

**Thank you for the comment. We make reference to the main study in the methods section where we reference the original study as per the statement below (page 4):**

**“Detailed enrolment and diagnosis criteria have previously been described (17). Briefly, persons who had a history of prior TB treatment and were suspected of having drug-resistant pulmonary TB were recruited into a prospective cohort study.”**

**“Presumptive drug-resistant TB patients were identified as symptomatic patients presenting with a history of >=1 month of prior TB treatment (relapse, treatment after loss to follow up or treatment failure), contact with a person with known or possible drug-resistant TB; or with a rifampicin-resistant result on Xpert MTB/RIF. RR-TB diagnosis was confirmed by molecular TB assay and/or phenotypic drug susceptibility testing.”**

Table 1: Many variables on this table don’t add up to 68: e.g. age categories, occupation etc.

**We have corrected this by adding information as a footnote at the bottom of the table to show number of patients with missing information for each variable.**

***Missing Information**

**HIV status, n=2; Education, n=1; Occupation, n=1; TB history, n=1; Prior TB incidents, n=1**

Page. 9, L161; This information needs to be reflected in the abstract

**Thank you. We have added this information to the Abstract as requested.**

Page. 9, L164; Participants who did not first care at**…..**

**corrected**

Page. 9, L168: Qualitative data collection and analysis was not described in the methods, how come these results?

**Thank you for this comment. We have added the below statements to the methods section to describe how qualitative data was captured (pages 4-5) and analysed (page. 5 and 6) in the study.**

**“Data were collected using a mixed method approach via an open-ended healthcare-seeking behaviour questionnaire designed to capture both quantitative and qualitative responses. A trained research nurse administered the questionnaire.”**

**“Thematic analysis was used to identify and analyse patterns and trends within the qualitative data gathered from the open-ended questionnaires.”**

Page. 9, L176: Figure legend present but figure absent

**Figure 2 was previously submitted separately as per the author guidelines but has now been added to the main document as well.**

How does the information presented in L143 differ from L184

**Thank you for this comment. In L143, we summarised the proportion of patients visiting different facilities, in L184, we summarised the combined facility visits.**

Page. 9, L183: Remove space between visits and recorded-

**corrected**

Page.9, L186: Figure legend present but figure absent

**Figures were submitted separately as per the author guidelines but have now been added to the manuscript as well.**

Page.9, L190: This should have been defined under methods. Pls add clinical outcomes to the definitions. How many people died or survived?

**Thank you. We have replaced the phrase “penultimate” with final and have added the below statement to the methods section (page 5) and the definitions section:**

**“**Final clinical outcomes were defined as 24-month patient survival and was ascertained through review of medical records and contact with next-of-kin.**.”**

**Based on this definition 6/68 (9%) patients died while 62/68 (91%).**

Page. 11, L212: contacted**-**

**corrected**

Page.11, L233, 236 : Integrate ref 5 and 29 as one statement as both sentences refer to the same thing-

**corrected**

Page. 12, L241: for a bedridden**….-**

**corrected**

Page. 12, L254: or by exorbitant**….-**

**corrected**

L257: another recommendation could be integrating private providers in care provision given their high patronage

**Thank you for the suggestion, which we have incorporated into our discussion. We have added the following statement to the discussion/conclusions (page. 13-14):**

**“As pharmacies are the first point of contact for many patients, we recommend that pharmacists and other private health care providers should be trained in appropriate TB diagnosis and referral of potential TB patients to hasten access to treatment and reduce the cyclic pathways evident in settings such as ours.”**

L264; use because instead of to the extent that-

**corrected**

L271, 272: Interviews not discussed in methods section

**Thank you. We have corrected this and added this information in the methods section as below (page 5):**

**“Data were collected using a mixed methods approach via an open-ended healthcare-seeking behaviour questionnaire designed to capture both quantitative and qualitative responses through patient interviews. The open-ended questionnaire allowed respondents to provide detailed responses on their health-seeking pathways and reasons for delay in seeking care. A trained research nurse administered the questionnaire.”**

L275: self-report is another limitation

**We appreciate this suggestion and have added the following statement to the limitations section (Page 13):**

**“Furthermore, data was self-reported and so may be subject to self-report bias.”**

L281: not discussed in the discussion, see comment above

**Thank you for this comment. We have corrected this and added more information about patient interviews in the methods section (page. 5):**

**“Data were collected using a mixed methods approach via an open-ended healthcare-seeking behaviour questionnaire designed to capture both quantitative and qualitative responses through patient interviews. The open-ended questionnaire allowed respondents to provide detailed responses on their health-seeking pathways and reasons for delay in seeking care.**

L284: We recommend the improvement of the quality of care………in order to reduce……….and ensure more prompt**……………**

**-corrected**

Reviewer #2: PONE-D-20-13834
Health care seeking patterns among patients investigated for drug resistant tuberculosis in Harare, Zimbabwe: a prospective cohort study
Thank you for a really interesting and important piece of work. Suggestions below are merely to help you strengthen your work and the final product. I have added comments with reference to specific sections of the work below.
Abstract:
Would be good if you could specify what your data source is; as well as outline your study design which I think is a mixed methods study (cross-sectional quantitative data) with qualitative interviews?
**We have added the below statement to the methods- Data collection section (page 4-5):**

**“We conducted a prospective cohort study……”**

**We have also added the information:**

**“Data were collected using a mixed method approach via an open-ended healthcare-seeking behaviour questionnaire designed to capture both quantitative and qualitative responses through patient interviews. A trained research nurse administered the questionnaire.”**

General comments:
Please define all abbreviations used in your manuscript.

**Abbreviations have been checked and defined fully at first mention in the manuscript.**

Page 3, line 64: I think you mean disability, instead of debility……..

**corrected**

Page 4, line 74: how many facilities were sampled and how were these facilities selected?
**We have clarified this under methods section as follows (page 4):**

**“….Patients were recruited from eight conveniently selected health facilities.”**

Page 4, line 87: should read “data were collected…” –

**corrected**

Page 4, line 91: where were the other variables collected from if not directly from the patient through patient interviews?-

**All data were collected from patient interviews. We have replaced “Other” with additional in this sentence and added the following text (page 5):**

**“Data were collected using a mixed methods approach via an open-ended healthcare-seeking behaviour questionnaire designed to capture both quantitative and qualitative responses through patient interviews. The open-ended questionnaire allowed respondents to provide detailed responses on their health-seeking pathways and reasons for delay in seeking care.”**

Page 5: under your statistical analysis paragraphs, it might be useful to specify different types of analyses separately. So, for example, perhaps start with describing how you would do your initial descriptive analysis where you are describing your sample. Specify if you are comparing different types of patients in your sample with one another. Then describe how you are setting up your regression analysis where you are wanting to explain your findings related to the time spent in accessing care or you are wanting to understand which variables and how they explain patient and health seeking delays. Then explain which statistical approaches you used and why.

**Thank you for this suggestion we have incorporated and added details of statistical methods in the statical analysis section (page 6):**

**“Descriptive statistics for numeric variables (such as age or income) were computed and are reported as median and interquartile range (IQR). Chi-square tests were used to compute associations between continuous and categorical variables. We used the Kruskal Wallis test to analyse the associations between health care facility first attended;”**

**“Both simple and multiple linear regression analyses were used to assess the associations between delay and explanatory variables. Potential risk factors for delay and variables associated with delay in the bivariate analysis (*P* ≤0.2) were included in a final multiple linear regression model.”**

**“ Thematic analysis was used to identify and analyse patterns and trends within the qualitative data gathered from the open-ended questionnaires (19).”**

Page 5, line 106: Why did you categorise your continuous variables, was that for the initial reporting in Table 1? In your statistical analysis, it is better if you can use the continuous variables in your model.

**Categorisation of data was only done for the initial reporting in Table 1. For the rest of the analyses, including the multiple linear regression, continuous variables were used.**

Page 5, line 124: It would be helpful if you could report not only the percentage, but to also show the numerator and denominator for the percentage reported, ex. 45% (31 /68) of participants had a history of TB.

**Thank you, the suggested changes have been incorporated the suggested changes throughout.**

On page 6, line 126: How do you define informal employment. It is worth spelling this out a bit more for the reader.

**We have added our operational definition of informal employment to the list of definitions as follows (page. 6):**

**“Informal employment was defined as engagement in an economic activity that is not taxed or formally registered.”**

Page 6, line 128: Figure 1 is not showing up in the review document. What were the reasons for patients to not consent to the health seeking behaviour survey? How did your sample reduce from 73 following initial consent to 68 included in your analysis?

**All figures were submitted separately as per author guidelines.**

**Out of the 73 patients, five patients did not have complete follow-up data and were excluded from the analysis. We explain this in the results section under “study participants” and have provided further clarity in the text as follows (page 7):**

**“A final sample of 68 participants with follow-up data were included in the final analysis; five participants were lost to follow up during the study period and were excluded from further analysis.”**

Table 1:
High proportion of the sample was female – why is this? Is it a possible sampling bias?

**We have acknowledged this bias and have added the following limitation and reference in the manuscript (page. 14-15):**

**“Third, female participants were over-represented in our study, (17) indicating a possible selection bias.”**

What is the relevance of having been referred from outside Harare? A more detailed description of the setting and DR-TB services in Harare will be needed in your background so that this statement makes sense. Plus, you need to remember that your readers may not know what the health system in Zimbabwe is like, and how people move through care. How do they pay for care? In your introduction, it would strengthen your paper if you could share some of your knowledge of how the system works to give the reader a sense of why your research question is important and what the context is within which they need to understand the results.

**Thank you for this comment. We have tried to clarify the context of our study by adding the following text in our methods (study population) section (page. 4):**

**“At the time our study was undertaken, a partially decentralized MDR-TB/RR-TB treatment system was in place in Harare. Patients could only be diagnosed and recommended for treatment via a central MDR-TB clinic (i.e., at Wilkins Hospital) based in Harare, but MDR-TB/RR-TB treatment could be dispensed through peripheral, local health clinics.”**

When you describe the delays; it would be good to first describe patient delay (as you have done), then health facility delay and then the total delay. This allows the reader to follow how you got to your total delay estimate.
**Thank you for this suggestion. We have spelled out our definitions of delay in the definitions section which precedes the statistical analysis (page 5) as follows:**

**“Patient delay was defined as the time from onset of disease-associated symptoms to first health care visit. Health system delay was defined as the time from first medical facility visit to initiation of effective RR-TB treatment. Total delay was defined as the sum of patient and health system delays and included both diagnostic delay and repeat visits before initiation of treatment.”**

Do you perhaps have any data on what proportion of people first started drug-sensitive TB treatment (DS-TB) before initiating on rifampicin resistant TB treatment?

**Thank you for this important question. Unfortunately we do not have this data as our study was not designed to answer this question.**

Page 9, line 161: the results here sounds as if they came from open-ended questions/ or from qualitative work. If so, you need to be clear with the reader where that data came from and how it was analysed. This needs a separate section in the methods section with detailed discussion and justification of the methods used.-

**Thank you for the opportunity to clarify this. We have added the following detail in the methods section (page 5):**

**“Data were collected using a mixed methods approach via an open-ended healthcare-seeking behaviour questionnaire designed to capture both quantitative and qualitative responses through patient interviews. The open-ended questionnaire allowed the respondents to provide detailed responses on their pathways and reasons for delay in seeking care. A trained research nurse administered the questionnaire.”**

**“Thematic analysis was used to identify and analyse patterns and trends within the qualitative data gathered from the open-ended questionnaires (18).”**

Page 9, line 174: Figures 2 and 3 are missing

**Figures were supplied as a separate document as per the author guidelines but have now been added to the main document as well (page 25-27).**

In the section “Associations with clinical outcomes” you are reporting important and interesting results. But I think this part of your analysis needs to be more clearly described in your methods section – specifically here I am referring to your comparison of costs and health seeking delays between who die and those who survive. How was this outcome (death) collected and what are the limitations of the approach used?

**We have added the following statement to the methods (page 5):**

**“Final outcomes were ascertained through review of medical records and contact with next-of-kin.”**

Next, how did you set up your regression analysis and then in the results even if those results are not significant it would still be useful to know what the direction of the relationship between those variables is and even if not significant you can still report the p-value. It would strengthen your paper if you could explore what the determinants of long delays to RR-TB treatment are… for example, it might be accessing a certain facility first, or age or gender… But if you set up your regression analysis to answer this question, the answers would be very helpful to policy makers as it would allow them to construct useful and data-informed interventions.

**Thank you for this comment. We have added some additional detail to the methods- (statistical analysis) section as below (Page.6-7):**

**“Both simple and multiple linear regression analyses were used to assess the association between delay and explanatory variables. The data for total delay were skewed. Potential risk factors for delay, and variables associated with delays in the bivariate analysis (*P* ≤0.2), were included in the final multiple linear regression model. A p-value of less than 0.05 was considered statistically significant. R software (version 4) was used to compute the visual pathways of care. Thematic analysis was used to identify and analyse patterns and trends within the qualitative data gathered from the open-ended questionnaires.”**

**Further we have added the following text to the results section (page10-11):**

**“We found that after adjusting for other variables, living with HIV and being employed were associated with shorter total delay, (p=0.11, median 98 days and p=0.25, median 104 days respectively), and TB retreatment was likely to be associated with a longer delay (p=0.20). We found no significant associations with mortality and median total delay or total cost incurred (Table 3). However, our sample size limited our ability to make conclusive determinations about the associations between delay and the risk factors that were assessed.”**

**We have also added Table 3 as shown below (Page. 22):**

**Table 3: Association between delay, cost and 24-month survival**

|  | **Died (n=6)** | **Survived (n=62)** | **p-value*** |
| --- | --- | --- | --- |
| Total delay (days) | 168.5 (83.3-309.25) | 132.0 (49.8-280.8) | 0.77 |
| Patient delay (days) | 30 (23.3-30) | 21 (14-42) | 0.62 |
| Total cost (dollars, US) | 24.5 (8-40.3) | 21.5 (10.3-55.5) | 0.75 |

*Mann-Whitney test

Your discussion section is great – the work is well positioned. The paper would be even stronger if you added a sentence or two on what your think the policy implications of the work are. What is your advice to the NTP, how should they use your results to improve the service?

**Thank you for the suggestion. We have added the following additional recommendations (page 13-14):**

**“As pharmacies are the first point of contact for many patients, we recommend that pharmacists and other private health care providers should be trained in appropriate TB diagnosis and referral of potential TB patients to hasten access to treatment and reduce the cyclic pathways evident in settings such as ours.”**
