# Supplementary material for: Health care seeking patterns of rifampicin-resistant tuberculosis patients in Harare, Zimbabwe: A prospective cohort study
Source: PLoS One. 2021 Jul 16;16(7):e0254204. doi: 10.1371/journal.pone.0254204 (PMC8284678; doi:10.1371/journal.pone.0254204)
Supplement: Attachment — Submitted filename: Responses to reviewers_17May2021.docx [file pone.0254204.s003.docx]

Authors’ Responses to Reviewer's Questions **are in dark font**.

**Comments to the Author**

4. Have the authors made all data underlying the findings in their manuscript fully available?

The [PLOS Data policy](http://www.plosone.org/static/policies.action#sharing) requires authors to make all data underlying the findings described in their manuscript fully available without restriction, with rare exception (please refer to the Data Availability Statement in the manuscript PDF file). The data should be provided as part of the manuscript or its supporting information or deposited to a public repository. For example, in addition to summary statistics, the data points behind means, medians and variance measures should be available. If there are restrictions on publicly sharing data—e.g., participant privacy or use of data from a third party—those must be specified.

Reviewer #2: No

**We have submitted our dataset as per PLOS Data policy and as requested in the previous review (File named: Anonymized Study Data).**

Review Comments to the Author

Reviewer #2: PONE-D-20-13834R1

My comments were sufficiently addressed. Additional comments, with further information:

In Table 2: show days per unit of time.

**We have corrected Table 2 and added the units (days) as suggested by the reviewer.**

Patient population: Rif-resistant.

**We have also corrected the table heading to include the patient population description, “RR-TB Patients’’. (page 21)**

Page 2, from line 35: better to quote the arithmetic mean of costs (in addition to the median). Cost data are typically right skewed. The arithmetic mean is used when we estimate the total cost to a budget (mean cost x number of patients to be treated). This is a common use of health service cost data when considering policy options.
A paper of interest is Thompson & Barber. 2000. How should cost data in pragmatic randomised trials be analysed? <https://www.ncbi.nlm.nih.gov/pmc/articles/PMC1127588/>.

**Thank you for this suggestion. We have amended this sentence to read “at a median cost of 13% (IQR 6-31%) of their total annual household income (mean cost, US$410” (line 32-33).**

**Likewise, we have also amended line 172-173 to read “Overall, a median of 13% (IQR 6-31%) or mean cost of US$410 of total annual household income”.**

Page 3, line 72: prospective cohort study nested in what?

**We have amended this sentence to simply read “We conducted a prospective cohort study….” (line 72)**

Page 9, line 202: would be good to specify the variables you adjusted for

**We have amended this statement to read “We found that after adjusting for age and gender....” (line 203)**

Page 9, line 217 and page 8, line 173: costs as a percentage of annual income is shown. It would be helpful if you could also report the proportion of patients who faced catastrophic costs. This will bring your paper in line with current literature. For an overview of the topic, see <http://www.stoptb.org/wg/dots_expansion/tbandpoverty/assets/documents/Tool%20to%20estimate%20Patients%27%20Costs.pdf>.

**We have added the following statement : “At least 30% (23/68) households incurred TB related costs which can be considered as catastrophic (12), i.e., above 20% of annual household income.” (line 175-176)**

**We have also amended the discussion section to include the statements: “Patient costs averaged approximately 13% of monthly income with at least 30% of the households incurring costs that may be considered “catastrophic” “(line 220-221)**

**“Up to 30% of households reportedly spent more than 20% of annual household income on TB related costs, a threshold which has been previously described as catastrophic (12).” (line 264-266)**
