# Supplementary material for: Health care seeking patterns of rifampicin-resistant tuberculosis patients in Harare, Zimbabwe: A prospective cohort study
Source: PLoS One. 2021 Jul 16;16(7):e0254204. doi: 10.1371/journal.pone.0254204 (PMC8284678; doi:10.1371/journal.pone.0254204)
Supplement: Attachment — Submitted filename: Responses to reviewers_28May2021.docx [file pone.0254204.s004.docx]

Authors’ Responses to Reviewer's Questions **are in dark font**.

**Comments to the Author**

Additional Editor Comments (if provided):

The team has done an excellent job with revisions. The manuscript looks quite good; I am excited to see this published.

I have one small suggestion for your consideration:

Line 304 (concluding paragraph): please consider revising by inserting the word 'screening' such that the revised text states "should be trained in appropriate TB screening and diagnosis, and referral of potential TB patients...."

The addition of the addition of 'screening' is perhaps more appropriate for the pharmacy level of care.

**We have revised the sentence to read “………we recommend that pharmacists and other private health care providers should be trained in appropriate TB screening and referral of potential TB patient………” as suggested by the Editor.**
